# The Fungal Cell Wall: *Candida*, *Cryptococcus*, and *Aspergillus* Species

**DOI:** 10.3389/fmicb.2019.02993

**Published:** 2020-01-09

**Authors:** Rocio Garcia-Rubio, Haroldo C. de Oliveira, Johanna Rivera, Nuria Trevijano-Contador

**Affiliations:** ^1^Center for Discovery and Innovation, Hackensack Meridian Health, Nutley, NJ, United States; ^2^Instituto Carlos Chagas, Fundação Oswaldo Cruz (Fiocruz), Curitiba, Brazil; ^3^Division of Infectious Diseases, Department of Medicine, Albert Einstein College of Medicine, New York, NY, United States

**Keywords:** cell wall, *Candida*, *Cryptococcus*, *Aspergillus*, synthesis, composition

## Abstract

The fungal cell wall is located outside the plasma membrane and is the cell compartment that mediates all the relationships of the cell with the environment. It protects the contents of the cell, gives rigidity and defines the cellular structure. The cell wall is a skeleton with high plasticity that protects the cell from different stresses, among which osmotic changes stand out. The cell wall allows interaction with the external environment since some of its proteins are adhesins and receptors. Since, some components have a high immunogenic capacity, certain wall components can drive the host’s immune response to promote fungus growth and dissemination. The cell wall is a characteristic structure of fungi and is composed mainly of glucans, chitin and glycoproteins. As the components of the fungal cell wall are not present in humans, this structure is an excellent target for antifungal therapy. In this article, we review recent data on the composition and synthesis, influence of the components of the cell wall in fungi-host interaction and the role as a target for the next generation of antifungal drugs in yeasts (*Candida* and *Cryptococcus*) and filamentous fungi (*Aspergillus*).

## Introduction

The fungal cell wall is an essential structure with great plasticity that is vital to maintaining cellular integrity and viability. The cell wall plays an important role in different biological functions such as controlling cellular permeability and protecting the cell from osmotic and mechanical stress (Ponton, 2008; Gow et al., 2017; Agustinho et al., 2018). In addition to these important functions, the cell wall mediates interactions with the external environment through adhesins and a large number of receptors that, after their activation, will trigger a complex cascade of signals inside the cell (Ponton, 2008). The cell wall is uniquely composed of polysaccharides and proteins as well as lipids and pigments (Gow et al., 2017). Furthermore, some wall components are very immunogenic and stimulate cellular and humoral responses during infection (Erwig and Gow, 2016). β-glucans and mannans, as well as antibodies directed against them, are very useful diagnostic tools since they can be detected in patients with invasive fungal infection (Pazos et al., 2006). As mentioned above, the cell wall represents an indispensable structure, that its disruption can have serious effects on cell growth and morphology resulting in cell death. Hence, it is considered a good antifungal target (Heitman, 2005; Cortes et al., 2019).

The cell wall is a specific and complex cellular organelle composed of glucans, chitin, chitosan, and glycosylated proteins. Proteins are generally associated with polysaccharides resulting in glycoproteins. Together, these components contribute to the cell wall rigidity. The synthesis and maintenance of cell wall involves a large number of biosynthetic and signaling pathways (Casadevall and Perfect, 1998).

In the following sections, the different components of the fungal cell wall will be reviewed generally and then, specifically focused on three fungi species, *Candida albicans*, *Cryptococcus neoformans*, and *Aspergillus fumigatus*. The characteristics of their components, their relationship with virulence, pathogenicity, and the interaction with the host’s immune system are reviewed. We also mention different works in which different components of the cell wall are possible targets for antifungal therapies. Recently, it has been proposed that the cell wall is particularly important in biotechnology to develop new antifungal drugs as well as inhibitors of certain cell wall components that are being tested in clinical trials. For a review on this topic, see reference (Cortes et al., 2019). The fungal cell wall is an extensive and complex topic and we highlight critical literature, but it is not possible to cite every study.

## Cell Wall Structure

The cell wall is structured in different layers where the innermost layer is a more conserved structure on which the remaining layers are deposited and can vary between different species of fungi. The composition and organization of fungal cell walls are compared and contrasted in the text below.

### Glucans

Glucan is the most important structural polysaccharide of the fungal cell wall and represents 50–60% of the dry weight of this structure. Most polymers of glucan are composed of 1,3 linkage glucose units (65–90%), although there are also glucans with β-1,6 (in *Candida* but not in *Aspergillus*), β-1,4, α-1,3 and α-1,4 links. The β-1,3-D-glucan is the most important structural component of the wall, to which other components of this structure are covalently linked. The β-1,3-D-glucan is synthesized by a complex of enzymes located in the plasma membrane called glucan synthases. The genes encoding β-1,3-D-glucans, *FKS1* and *FKS2*, were initially identified in *Saccharomyces cerevisiae* (Douglas et al., 1994; Qadota et al., 1996; Ponton, 2008). Analogs of these genes are currently known in several species of *Candida*, *Aspergillus*, *Cryptococcus*, and *Pneumocystis* among other fungi. Disruption of one of these genes affects cell growth (Douglas et al., 1994; Mazur et al., 1995) but elimination of both causes cell death (Mazur et al., 1995; Bowman and Free, 2006). The α-1,3-glucan is also a fundamental component of the fungal cell wall and is synthetized by α-glucan synthase (*AGS1*).

### Chitin

The chitin content of the fungal wall varies according to the morphological phase of the fungus. It represents 1–2% of the dry weight of yeast cell wall while in filamentous fungi, it can reach up to 10–20%. Chitin is synthesized from n-acetylglucosamine by the enzyme chitin synthase, which deposits chitin polymers in the extracellular space next to the cytoplasmic membrane. The of chitin content in the *C. albicans* hyphae wall is three times higher than that of yeasts (Chattaway et al., 1968) while the chitin content of the mycelial phases of *Paracoccidioides brasiliensis* and *Blastomyces dermatitidis* is 25–30% of that yeast phase (Kanetsuna et al., 1969).

### Glycoproteins

Proteins compose 30–50% of the dry weight of fungal wall in yeast and 20–30% of the dry weight of the wall of the filamentous fungi. Most proteins are associated to carbohydrates by O or N linkages resulting in glycoproteins. Cell wall proteins have different functions including participation in the maintenance of the cellular shape, adhesion processes, cellular protection against different substances, absorption of molecules, signal transmission, and synthesis and reorganization of wall components (Bowman and Free, 2006; Ponton, 2008).

### Melanin

Melanin is a pigment of high molecular weight that is negatively charged, hydrophobic and insoluble in aqueous solutions and protects fungi against stressors facilitating survival in the host (Liu et al., 1999; Casadevall et al., 2000; Nosanchuk and Casadevall, 2006; Nosanchuk et al., 2015). The fungi produce melanin by two routes, from 1, 8-dihydroxynaphthalene (DHN) intermediate and from L-3, 4-dihydroxyphenylalanine (L-dopa) (Eisenman and Casadevall, 2012). Melanin production contributes to fungal virulence (Salas et al., 1996; Noverr et al., 2004; Silva et al., 2009), improves resistance to environmental damage such as extreme temperature, UV light and toxins (Rosa et al., 2010; Zalar et al., 2011; Eisenman and Casadevall, 2012), and is important for invasion and dissemination. For example, *C. neoformans* melanin has been linked with dissemination of yeast cells from the lungs to other organs (Noverr et al., 2004), is known to influence the immune response of the host (Eisenman and Casadevall, 2012) and inhibit phagocytosis (Wang et al., 1995). In *Aspergillus*, melanin inhibits macrophage apoptosis that have phagocytosed melanized conidia (Volling et al., 2011).

## Candida albicans

*Candida* species are part of the mucous flora and can cause a broad spectrum of human infections. This genus includes at least 30 species of clinical importance (Pfuller et al., 2011; Silva et al., 2012). During the last decades, the incidence of infections caused by *Candida* genus has increased significantly (Sobel, 2007; Pfuller et al., 2011). *C. albicans* is the species that is most frequently isolated in cases of candidiasis (45–50%) (Del Palacio et al., 2009).

### Composition and Biosynthesis

*Candida albicans* is the most common opportunistic pathogen and cause of invasive fungal infection in hospitalized patients (Sobel, 2007; Pfuller et al., 2011). It is a highly adaptable fungal species with a large repertoire of virulence factors that allows its transition from commensal organism to pathogen. Thus, one of the key virulence characteristics is its ability to switch morphologies between yeast cells, pseudohyphae, and hyphae (Tsui et al., 2016). The main difference between the yeast and the hyphal form is that the hyphal wall has a slightly higher chitin content than the yeast form (Braun and Calderone, 1978). In addition, the structure of cell wall mannans differs between morphotypes, with a significant decrease in phosphodiesterified acid-labile β-1,2-linked manno-oligosaccharides in the hyphal form, whereas the amount of acid-stable β-1,2 linkage-containing side chains remains the same (Shibata et al., 2007).

**FIGURE 1:**
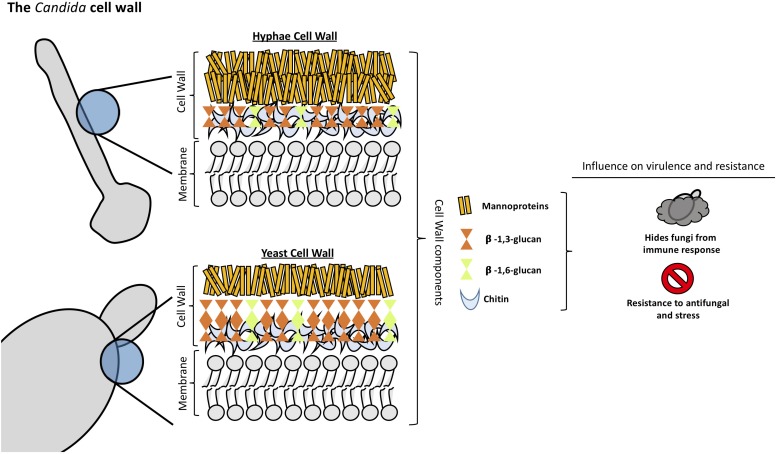
Structural organization and composition of *Candida albicans* cell wall.

*Candida albicans* cell wall is a two-layered structure. The main core of the cell wall is composed of a β-glucan-chitin skeleton, which is responsible for the strength and shape of the cell wall (see Figure 1). Chitin is located in the inner layer of the cell wall (Gow and Hube, 2012) and its chains can form tight antiparallel hydrogen-bonded structures associated with high insolubility (Chaffin, 2008). In *C. albicans*, there is one *CHS* family composed of four genes. It has been described that *CHS1* from class II is an essential chitin synthase and is involved in septum formation, viability, cell shape and integrity (Munro et al., 2001).

As in other fungi, the most abundant molecules in *C. albicans* are β-1,3-glucans. They are in the inner cell wall linked to β-1,6-glucans, which connect the inner and outer cell wall (Brown and Gordon, 2005). β-1,3-glucan synthases are responsible for the synthesis of β-1,3-glucans and consist of an enzyme complex with at least two subunits, Fksp and Rho1p. In *C. albicans*, Fksp is encoded by three ortholog genes, *FKS1*, *FKS2*, and *FKS3*, which catalyzes the transfer of sugar moieties from activated donor molecules to specific acceptor molecules forming glycosidic bonds (Sawistowska-Schroder et al., 1984).

β-1,6-glucans are side chains of variable lengths and distributions that can form complex structures stabilized by interchain hydrogen bonds. They act as a linker molecules binding different cell wall proteins to the β-1,3-glucan-chitin core through glycosylphospatidyl inositol (GPI) proteins (Klis et al., 2001). β-1,6-glucan synthase has not been identified in any fungal species, however, several genes that affect the synthesis of this compound have been described in *S. cerevisiae* (Lesage and Bussey, 2006). Interestingly, *C. albicans* cell wall contains considerably more β-1,6-glucan compared to *S. cerevisiae* due to either an increase in the number of molecules or an increase in glucose residues, or both (Brown and Gordon, 2005). Unlike *Aspergillus* or *Cryptococcus* spp., α-(Ponton, 2008; Gow et al., 2017)-glucan is absent from *Candida* spp. cell wall (Yoshimi et al., 2017).

The outer layer of *C. albicans* cell wall is packed with mannoproteins that are glycosylphosphatidylinositol (GPI)-modified and cross-linked to β-1,6-glucans (Shibata et al., 2007). N-linked mannans are composed of α-1,6-mannose backbone with α-1,2-oligomannose sidechains capped with β-1,2- mono-, di-, tri-, or tetra mannans (Shibata et al., 2007). O-linked mannans are found associated with cell wall glycoproteins. Some protein mannosyltransferases are responsible for the first steps in the O-linked mannans biosynthesis, adding a mannose residue to a serine or threonine residue. Additional mannoses are added by α-1,2-mannosyltransferases which results in a short α-1,2-mannose chain. The last step consists of the addition of an α-1,3-mannoses by α-1,3-mannosyltransferases (Free, 2013).

Mannans are less rigid compared to β-glucans and chitin, so they do not influence the cell shape. However, they have low permeability and porosity which affect the resistance of the cell wall to antifungal drugs and host defense mechanisms (Gow and Hube, 2012). Furthermore, since the outer mannan layer covers the inner layers of the cell wall, it has been described to be important in immune evasion concealing β-glucans from host immune detection (Hernandez-Chavez et al., 2017). Mannans are considered pathogen associated molecular pattern (PAMP) ligands and many host receptors are known to participate in its recognition (Brown et al., 2002; Rubin-Bejerano et al., 2007). *Candida glabrata* contain mannans with a structure closely resembling *S. cerevisiae* mannans since it is genetically more closely related to this species (Kobayashi et al., 1998). In addition, *C. glabrata* cell wall has 50% more protein and a higher mannose/glucose ratio than *S. cerevisiae* walls (de Groot et al., 2008; Lima-Neto et al., 2011).

### Influence of *Candida* Cell Wall Components on Fungi-Host Interaction

Fungi cell wall plays an essential role in the interaction with host cells and tissues. The components of the cell wall are of great importance in protecting the fungi, shifting the host immune response in favor of fungal growth allowing dissemination in the host (Poulain and Jouault, 2004; Galan-Diéz et al., 2010; Sem et al., 2016). β-glucan is easily recognized by the host immune system producing an effective response against the infection and thereby, protecting the host. Hence, masking of β-glucan is one of the most important mechanisms of *Candida* species and any disturbance of the synthesis and organization of the cell wall components results in the unmasking of the glucan layer increasing the capacity of the host immune system to recognize and attack the fungi pathogen (Granger, 2018).

Mannoproteins form a fibrillar layer containing O-glycosylated oligosaccharide and N-glycosylated polysaccharide moieties of the most external *Candida* cell wall layer. Mannoproteins are essential in *Candida* interaction with the host allowing the activation and modulation of the immune response against the fungi (Gow and Hube, 2012; Shibata et al., 2012; Paulovicova et al., 2015). They mask the β-glucan layer decreasing the recognition of fungi by the host immune system a process that is mediated by dectin-1, impacting directly the capacity of the host phagocytic cells to uptake and kill *Candida* cells (Galan-Diéz et al., 2010; Bain et al., 2014). In addition, masking the β-glucan layer confers *C. albicans* resistance to complement activation, via classical and alternative pathway, leading to an ineffective activation of the host immune system (Zhang et al., 1997; Boxx et al., 2009, 2010). Ywp1 is an abundant mannoprotein in *C. albicans* cell wall. Mutant strains with disrupted *YWP1* gene resulted in increased exposure of β-glucan in the cell wall. The expression of this protein in germ tubes and hyphae leads to a decrease in the exposure of glucan molecules resulting in decreased glucan accessibility of those structures (Granger, 2018). MAPK signaling pathway was demonstrated by Galan-Diéz et al. (2010) to be involved in the process of β-glucan masking. They observed that the disruption of the *CEK1*-mediated MAPK pathway generates mutant strains with more exposure of the β-glucan layer in the cell wall, leading to an increase of the Dectin-1-mediated immune responses (Galan-Diéz et al., 2010).

Chitin plays an important role in the interaction of *Candida* species with the host. Chitin-deficient mutant strains display attenuated virulence in immunocompetent and immunosuppressed hosts even though these mutants are able to colonize distinct organs, revealing that the attenuated virulence profile is not due to accelerated clearing (Bulawa et al., 1995). Chitin can block the recognition of *C. albicans* by peripheral blood mononuclear cells (PBMCs) and murine macrophages leading to a significant decrease in cytokine production (Mora-Montes et al., 2011). In addition, an important feature of *C. albicans* cell wall chitin is its important role on arginase-1 induction in host macrophages generating alterations on macrophage nitric oxide production leading to a decrease on macrophage antimicrobial function (Wagener et al., 2017).

### Cell Wall as an Antifungal Target

The fungi cell wall is mostly composed of molecules that are not present in the human body and, therefore, constitute an ideal target for the development of clinical antifungal compounds and the design of immunotherapies.

Echinocandins drugs are antifungal compounds that target the β-1,3-glucan synthesis of the cell wall in a non-competitive way (Aguilar-Zapata et al., 2015). There are three commercially available drugs -caspofungin, micafungin, and anidulafungin- and a novel molecule with prolonged half-life -rezafungin (CD101)-, which is currently in phase 3 evaluation (Krishnan et al., 2017; Wiederhold et al., 2018).

Chitin is important for caspofungin resistance in some *Candida* species, such as of *C. albicans*, *C. tropicalis*, *C. parapsilosis*, and *C.guilliermondii*. It has been described that an increase in chitin content in some isolates of *C. krusei* as a consequence of caspofungin exposure (Walker et al., 2013). Strains with elevated levels of chitin in its cell wall also show an echinocandin resistant profile as revealed in a systematic *in vivo* infection model of candidiasis (Lee et al., 2012).

In addition, there is a novel drug called ibrexafungerp (SCY-078) that is a glucan synthase inhibitor belonging to the triterpenoid antifungal class and shows a broad *in vitro* and *in vivo* activity against a broad spectrum of *Candida* (Larkin et al., 2019). *In vitro* studies have demonstrated that this new drug has fungicidal activity against azole-resistant *Candida* spp. isolates similar to the echinocandins, but also against the majority of echinocandin resistant clinical isolates due to *FKS* gene mutations (Scorneaux et al., 2017).

## Cryptococcus neoformans

*Cryptococcus neoformans* is the etiological agent of the cryptococcosis, a systemic mycosis with dissemination to central nervous system causing meningoencephalitis and primarily affecting immunocompromised patients such as HIV-positive patients (Maziarz and Perfect, 2016; Rajasingham et al., 2017; Beardsley et al., 2019).

### Composition, Biosynthesis, and Interaction With the Host

**FIGURE 2:**
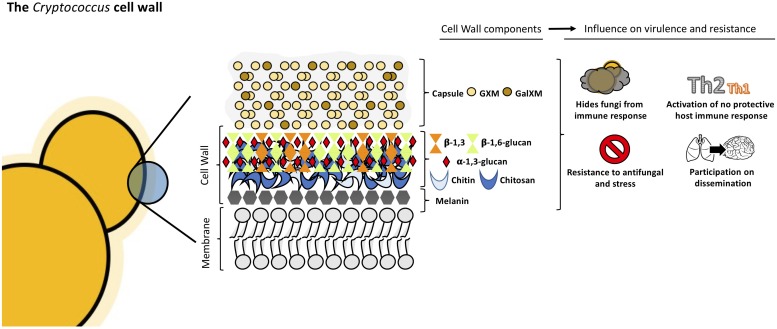
Structural organization and composition of *Cryptococcus neoformans* cell wall.

*Cryptococcus neoformans* cell wall is a dynamic structure that undergoes constant remodeling to modulate the distribution and crosslinking of its components necessary for cellular growth and division (Doering, 2009; Agustinho et al., 2018; Wang et al., 2018). *Cryptococcus* cell wall is a two-layered structure composed by α-1,3-glucan, β-1,3 and β-1,6-glucan, chitin, chitosan, mannoproteins and other GPI-anchored proteins (Baker et al., 2007; Doering, 2009; O’Meara and Alspaugh, 2012; Wang et al., 2018). The inner layer is mainly composed of β-glucans and chitin arranged as fibers parallel to the plasma membrane and the outer layer contains α-glucan and β-glucan (Sakaguchi et al., 1993; Doering, 2009; O’Meara and Alspaugh, 2012; see Figure 2). Collectively, these components are essential to maintain the cell shape and for the infection.

The exopolysaccharide capsule is anchored to the outer layer of the cell wall (O’Meara and Alspaugh, 2012; Wang et al., 2018) and this union needs to happen correctly since it is the main virulence factor of this yeast (Vecchiarelli, 2000; McFadden and Casadevall, 2001; Zaragoza et al., 2009). β-1,6-glucan is the most abundant component in *Cryptococcus* cell wall, while β-1,3-glucan is less abundant, contrary to other yeasts (Gilbert et al., 2010; Wang et al., 2018). The main functions of β-1,6-glucan are to maintain and organize the cell wall through interactions with other cell wall components contributing to the integrity of *Cryptococcus* cell wall. Genes such as *KRE5*, *KRE6*, and *SKN1* are involved in β-1,6-glucan synthesis and play an important role in maintaining proper growth, morphology and integrity of the cells (Zaragoza et al., 2009; Gilbert et al., 2010). Mutants for these genes are more sensitive to stress and displayed important alterations in the cell wall composition leading to the loss of virulence in mammalian host (Gilbert et al., 2010).

β-1,3-glucan is structural component of *Cryptococcus* cell wall. In other ascomycetes, β-1,3-glucan is the most abundant component but in *C. neoformans*, the percentage of β-1,3-glucan is lower (Casadevall and Perfect, 1998). β-1,3-glucan synthase gene (*FKS1*) is essential, indicating the importance of this conserved cell wall component (Thompson et al., 1999; O’Meara and Alspaugh, 2012). By activating FKS1, *Cryptococcus* is able to respond to stress by producing β-1,3-glucan (Wang et al., 2018). The inhibition of β-1,3-glucan synthesis induces cell death and changes in cellular morphology (Toh et al., 2017).

α-1,3-glucan is a fundamental component of cryptococcal cell wall and is synthetized by *AGS1*. If the *AGS1* gene is disrupted (*ags1*Δ strain), yeast cells remain alive but there is no capsule on the surface despite production of capsule components (Reese and Doering, 2003; Reese et al., 2007). This showed that α-1,3-glucan is important for the correct capsule-cell wall attachment in *C. neoformans*. In addition, α-1,3-glucan may be involved in protection against the immune system, acting as a shield, hiding the immunogenic β-glucans and chitin molecules, as shown in other pathogenic fungi such as *Histoplasma capsulatum*, *B. dermatitidis*, and *P. brasiliensis* (San-Blas and San-Blas, 1977; Rappleye et al., 2007; Koneti et al., 2008; O’Meara and Alspaugh, 2012).

Chitin is present in minor quantities in *C. neoformans* cell wall, nevertheless it contributes to the strength of the cell wall (Doering, 2009). In *Cryptococcus*, eight chitin synthases and three potential regulatory proteins coordinate and regulate chitin deposition in the cell wall (Banks et al., 2005; Doering, 2009). *CHS3P* is essential to cell integrity and its disruption leads to stress-sensitive cells which show morphological alterations and the inability to retain melanin (Banks et al., 2005; Wang et al., 2018). Chitin plays a crucial role in capsule architecture as revealed in chitin-like structures found in capsular material (Zaragoza et al., 2010). It has been shown that chitin of *C. neoformans* cell wall induces Th2-type immune response increasing the mortality of mice, demonstrating that chitin can modulate host immune system (Wiesner et al., 2015).

Chitosan, the deacetylated form of chitin, is also present in *C. neoformans* cell wall. Chitosan is a more soluble and flexible polymer (Doering, 2009) and the amount in the cell wall is three to five times higher than chitin. This ratio changes with cell wall density (Banks et al., 2005). *C. neoformans* encodes three chitin deacetylases genes, *CDA1*, *CDA2*, and *CDA3*. When these genes are disrupted, the mutants present with decreased chitosan levels which correlates with increased levels of chitin, defects in cell integrity, and increased capsule size (Baker et al., 2007; Doering, 2009). Fonseca et al. (2009) observed *in vitro* that chito-oligomers interfered in the *C. neoformans* capsule assembly. The addition of chito-oligomers to cultures of *C. neoformans*, resulted in aberrant capsules and interference with the connection of the capsule with the cell. Moreover, *in vitro* experiments in which *C. neoformans* chitin synthesis is inhibited by the addition of a glucosamine 6-phosphate synthase inhibitor resulted in capsules loosely connected to the cell wall and polysaccharide fibers with decreased diameter (Fonseca et al., 2009). Chitosan deficient strains displayed slow growth *in vivo* and attenuated virulence in mice model (Baker et al., 2011).

Chitosan mutants promote a protective Th1 host response (Upadhya et al., 2016) showing that chitosan is necessary for full virulence of *Cryptococcus*. An important structure of chitin is the amino sugar N-acetylglucosamine (GlcNAc). Recently, Camacho et al. (2017) showed that *C. neoformans* is able to metabolize exogenous GlcNAc as source of carbon and nitrogen. The supplementation of culture medium with GlcNAc lead to an increase in the chitin-to-chitosan levels. Collectively, the data suggests that *Cryptococcus* can use this exogenous GlcNAc to build its cell-walls and that GlcNAc influence on capsule structure and melanin deposition in the cell-wall.

Melanin is an important virulence factor of *C. neoformans* associated with the cell wall. This pigment is produced by laccase, confers resistance to stress factors, is immunogenic, modulates the host immune response and is known to play essential role in the dissemination of *Cryptococcus* to hosts brains (Liu et al., 1999; Mednick et al., 2005; Nosanchuk and Casadevall, 2006). Melanized *Cryptococcus* cells are less susceptible to amphotericin B and this phenotype may be due to modifications in the cell wall such as reduction of cell wall pores sizes resulting in melanized cells being considerably less porous than non-melanized yeast cells (Jacobson and Ikeda, 2005).

Finally, the components that complete the structure of *C. neoformans* cell wall are proteins that are embedded in the cell wall carbohydrates. Cryptococcal cell wall contains 29 GPI-anchored proteins, including proteases, carbohydrate-active enzymes and phospholipase B1 (Eigenheer et al., 2007). Phospholipase B1 (Plb1), which is covalently bound to β-1,6-glucan, is involved in membrane homeostasis, remodeling and maintenance of integrity of cell wall contributing to fungal survival in host environment and facilitating tissue invasion (Siafakas et al., 2007; O’Meara and Alspaugh, 2012). Plb1 mutants produced capsules of lower density, which may indicate its importance on capsule attachment to cell wall. In addition, these mutants demonstrated increased sensitivity to cell wall disturbing agents. Furthermore, the amount of Plb1 increases in the cell wall when in higher temperatures, suggesting the role of this protein in the defense of cryptococcal cells from temperature stress (Siafakas et al., 2007). Disruption of Plb1 in *Cryptococcus* results in attenuating its virulence as demonstrated by reduced fungal burden in murine infection models and decreased dissemination with a possible role in translocation through the blood-brain barrier (Santangelo et al., 2004; Chayakulkeeree et al., 2011; Maruvada et al., 2012; Evans et al., 2015).

Cryptococcal cell wall components are unique and are closely related with the capacity of this fungus to cause disease playing essential roles in response to different host and environmental stress (Wang et al., 2018). The capsule is the main virulence factor of *C. neoformans* (Vecchiarelli, 2000; McFadden and Casadevall, 2001; Zaragoza et al., 2009). As previously mentioned, cell wall components are key to proper capsule anchoring (O’Meara and Alspaugh, 2012). *C. neoformans* can increase its size two ways: increasing the size of the capsule which is widely studied (Zaragoza et al., 2008, 2009; Ding et al., 2016; Casadevall et al., 2018; Fonseca et al., 2018; Wang et al., 2018; Zaragoza, 2019) or increasing the size of capsule and cell body resulting in Titan cells, phenomenon less studied, resulting in cells which can reach 100 μM (Okagaki et al., 2010; Zaragoza et al., 2010; Garcia-Rodas et al., 2018). These studies suggesting cell wall re-modelation contribute to occurs during this morphological change. Titan cell formation results in thicker cell wall compared to normal cells (Zaragoza et al., 2010) composed with more glucosamine and less glucose, displaying less β-glucan, possessing in their outer cell wall layer α-glucans and structural mannans. Titan cells cell wall have increased chitin levels compared to normal size cells resulting in detrimental host immune response characterized by increased Th-2 type cytokines contributing to the disease progression in mice (Wiesner et al., 2015; Mukaremera et al., 2018). In addition, *in vitro* studies of Titan cells form thicker cell walls compared with “normal” cells with regular size suggesting cell wall re-modelation during this morphological change (Dambuza et al., 2018; Hommel et al., 2018; Trevijano-Contador et al., 2018).

### Cell Wall as an Antifungal Target

β-1,3-glucan synthase is a target for echinocandins compounds. However, while *FKS1* gene is essential in *Cryptococcus* and β-1,3-glucan synthase is sensitive to echinocandins *in vitro*, this antifungal is ineffective against *C. neoformans* infections (Maligie and Selitrennikoff, 2005; O’Meara and Alspaugh, 2012; Toh et al., 2017; Wang et al., 2018). Since internalization of echinocandins by *Cryptococcus* cells is necessary to inhibit β-1,3-glucan synthase, it has been hypothesized that *Cryptococcus* have an unknown mechanism that decreased the influx of the drug. However, this is still unclear and other mechanisms such as the inactivation of echinocandins by this yeast or other resistance mechanism are currently under investigation (Toh et al., 2017; Wang et al., 2018).

*Cryptococcus* cell wall is a dynamic structure that confers essentials tools necessary for the fungus to adapt to host environment. Yeast cells harbor an extensive molecular arsenal that act by protecting fungi from hosts and environmental stressors. *Cryptococcus* virulence factors such as capsule, formation of Titan cells and melanin are closely related to cell wall dynamics and composition highlighting the importance of the cell wall for *Cryptococcus* pathogenicity.

## Aspergillus fumigatus

*Aspergillus* spp. comprises a variety of environmental filamentous fungus found in diverse ecological niches worldwide and can cause life-threatening diseases in immunocompromised individuals with a wide range of clinical manifestations (Latge, 1999).

### Composition and Biosynthesis

Among this genus, *A. fumigatus* is the most prevalent species and is largely responsible for the increased incidence of invasive aspergillosis with high mortality rates in immunocompromised patients (Garcia-Rubio et al., 2017). Due to its clinical importance, this mold has become a model for studying filamentous fungus cell wall and understanding its role in growth and pathogenesis.

**FIGURE 3:**
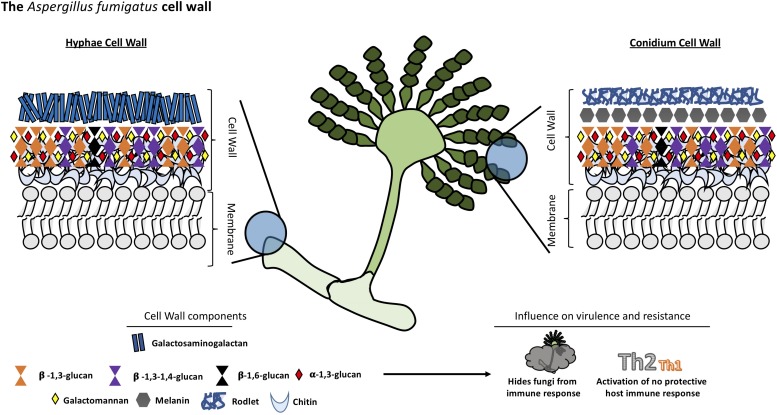
Structural organization and composition of *Aspergillus fumigatus* cell wall.

Like *Cryptococcus*, the cell wall of *Aspergillus* is a two-layered structure. In *Aspergillus*, the predominant cell wall components are polysaccharides synthesized by transmembrane synthases, transglycosidases and glycosyl hydrolases. The main core of *A. fumigatus* cell wall consists of a polymer of β-1,3-glucan and chitin which is responsible for the rigidity of this structure. β-1,3-glucan is cross-linked to α-1,3-glucan, galactomannan, galactosaminogalactan and a unique mixed molecule of β-1,3-1,4-glucans which has never previously described in fungi, all of them covalently bound one to the other (Fontaine et al., 2000). The composition of the outer cell wall varies between morphotypes, hyphae, and conidium which has a rodlet layer composed of hydrophobins followed by dihydroxynaphtalene melanin (Aimanianda et al., 2009; Bayry et al., 2014). Interestingly, there is no β-1,3-glucan nor chitin in the outer cell wall layer in contrast to other species (see Figure 3).

Chitin constitutes a much bigger fraction of the cell wall in filamentous fungi than in yeast, around 10–20% of the dry weight of cell walls. The external side of the membrane, the nascent chitin chain folds back on itself to form anti-parallel chains with intra-chain hydrogen bonds (Chantal et al., 2016). Multiple families of chitin synthases (CHS) are responsible for the synthesis of this compound and many isoforms have been identified bioinformatically. However, the specific function of each of them remains to be established. *A. fumigatus* is predicted to have eight CHS genes (Muszkieta et al., 2014). This multiplicity is conserved among many species and highlights the importance of chitin in fungi.

The other major cell wall component in *A. fumigatus* is β-1,3-glucan which is synthesized by a glucan synthase complex which contains two subunits, using UDP-glucose as a substrate. The catalytic subunit is encoded by *FKS1* gene, the target of echinocandins drugs. This gene is unique but non-essential in *A. fumigatus*. The Δ*fks1* deletion mutant showed a compensatory increase of chitin and galactosaminogalactan with a decrease in cell wall galactomannan (Dichtl et al., 2015). The *FKS1* protein is formed by 16 transmembrane helices and two external loops (Beauvais et al., 2001). The regulatory unit is a Rho1-GTPase encoded by *RHO1* gene, and it has been proposed to have a regulatory interaction between this subunit and the cell wall integrity pathway of *A. fumigatus* (Dichtl et al., 2012). The synthesis of other polysaccharides remains poorly understood. For example, α-1,3-glucan is an important cell wall component of *A. fumigatus* synthesized by three α-1,3-glucan synthases encoded by *AGS1*, *AGS2* and *AGS3* genes but the substrate of these enzymes is still unknown (Beauvais Anne and Latgé, 2006). Deletion of all the three *AGS* genes resulted in the lack of α-(Ponton, 2008; Gow et al., 2017)-glucan in the cell wall, and a decreased virulence in a murine model. However, its growth and germination was not affected (Beauvais et al., 2013).

Another integral component of the fungal cell wall in *A. fumigatus* is long linear chains of repeating mannan units formed of four α-1,6-linked and α-1,2-linked mannoses with side chains of galactofuran covalently bound to the chitin-glucan polysaccharide core. However, major differences have been found in the structural organization of the long mannans in yeasts, such as *S. cerevisiae* and *C. albicans*, compared to *A. fumigatus*. The highly branched mannans of these yeasts are linked to proteins but not covalently bound to the glucan-chitin core as has been found in *A. fumigatus* (Fontaine et al., 2000). Eleven putative mannosyltransferases have been detected in *A. fumigatus* as orthologous genes in yeast responsible for establishing α-1,6- and α-1,2-mannose linkages. However, the complete deletion of these genes did not lead to a reduction in the mycelial cell wall mannan content but caused a decrease in the mannan content of the conidial cell wall (Henry et al., 2016). Other orthologous genes of yeast mannosyltransferases, with a function not associated with mannan polymerization, were investigated and two members of the KTR family (also named Kre2/Mnt1) were found to be responsible for the polymerization of the structural cell wall galactomannan in this mold. Deletion of this gene led to a severe growth phenotype, a strong defect in conidiation, and a reduction of virulence in mouse models (Henry et al., 2019).

Various galactose-containing polymers are located in the *A. fumigatus* cell wall. The galactomannan is composed of mannan and galactofuranose and it is likely to involve a GPI anchor precursor (Costachel et al., 2005), while the galactosaminogalactan is composed of α-1-4 linked galactose and α-1-4 linked N-acetylgalactosamine residues (Fontaine et al., 2011). The presence of β-1,3-1,4-glucan in the *A. fumigatus* cell wall is a unique feature; it was the first description of this molecule in fungi (Fontaine et al., 2000). While this polysaccharide is a well-studied molecule in plants (Doblin et al., 2009), the role of this molecule in *A. fumigatus* is unknown, although, a study suggests one glycosyltransferase encoded by the *TFT1* gene (Three Four Transferase 1) is involved in the cell wall mixed linkage glucan synthesis (Samar et al., 2015). Once these linear, resynthesized polysaccharides are extruded into the cell wall, they have to be modified and cross-linked to one another resulting in the cell wall structural organization. In this context, some GPI-anchored transglycosidases have an important role in remodeling newly synthesized polysaccharides (Mouyna et al., 2013). For example, enzymes of the Gel family (GH72 family) are responsible for the elongation but also branching of the newly synthesize β-1,3-glucan (Gastebois et al., 2010; Aimanianda et al., 2017), while the DFG family takes part in the covalent binding of the galactomannan to the glucan-chitin core (Muszkieta et al., 2019).

### Host Immune Response to *Aspergillus fumigatus* Cell Wall Components

*Aspergillus fumigatus* releases abundant airborne conidia which are inhaled by humans. The first barrier involved in the *A. fumigatus* conidia clearance is formed by the airway mucociliary cells followed by the alveolar macrophages in the alveolar lumen before they undergo germination (Latge, 1999).

The composition of the cell wall varies depending on the stage of fungal growth, so the host’s immune response also varies (Lee and Sheppard, 2016). Dormant conidia have an outer layer formed of rodlets of RodA hydrophobins and dihydroxynaphthalene-melanin which are immunologically inert and mask the inner components of the fungi cell wall. Melanin is an important virulence factor for *Aspergillis* since it protects conidia from macrophages and epithelial cell phagocytic activity inhibiting acidification of phagolysosomes and phagocyte apoptosis (Amin et al., 2014; Bayry et al., 2014). After phagocytosis of conidia by alveolar macrophages and germination, the rodlets are degraded and the cell wall polysaccharides that were concealed become exposed, triggering a potent immune response.

The β-1,3-glucan is specifically recognized by a pattern-recognition-receptor (PRR), Dectin-1 (Herre et al., 2004) which is stimulated only by fibrillary or particulate forms of β-1,3-glucan but not by soluble forms. Dectin-1 is required for IL-23 production by dendritic cells and stimulating IL-17 production by neutrophils (Werner et al., 2009). It is also required for IL-22 responses as well asIL-1α, IL-12, CCL3, CCL4, and TNFα release (Gessner et al., 2012). Dectin-1 plays a role in the adaptive immune response to *A. fumigatus* whose deficiency results in altered specific-T cell maturation (Rivera et al., 2011) leading to an increased production of Dectin-1-dependent CXCL1, CXCL2, and TNFα by bone marrow-derived macrophages (Carrion Sde et al., 2013). These Dectin-1-dependent responses are more relevant in germinating conidia and young hyphae as higher levels of β-1,3-glucans are exposed than in mature hyphae where it is covered by exopolysaccharides (Gravelat et al., 2013). In relation to α-1,3-glucan, no host receptor has been identified. The triple deletion mutant of genes regulating biosynthesis resulted in an increased exposure of surface PAMPs, therefore it could play a role in masking those motifs from immune recognition (Beauvais et al., 2013).

One important exopolysaccharide is galactosaminogalactan, an adhesin that facilitates binding of hyphae to macrophages, neutrophils, and platelets (Fontaine et al., 2011; Rambach et al., 2015). It has been associated with an immunosuppressive activity masking cell wall β-glucans from recognition by Dectin-1, decreased polymorphonuclear neutrophil apoptosis via an NK cell-dependent mechanism and ROS production (Gravelat et al., 2013; Robinet et al., 2014). In addition, this polysaccharide promotes fungal development in immunocompetent mice due to its immunosuppressive activity associated with diminished neutrophil infiltrates (Fontaine et al., 2011). In humans, the polysaccharide inhibits Th1 and Th17 protective response toward Th2, promoting IL-1Ra secretion by human peripheral blood mononuclear cells (Gresnigt et al., 2014). Galactomannan has also a detrimental effect in the immune system favoring fungal infection. DC-SIGN is an adhesion receptor that specifically interacts with *A. fumigatus* cell wall galactomannans (Serrano-Gomez et al., 2004). Dectin-2 is another receptor that recognizes α-mannans and has an important role in conidia and hyphae binding by THP-1 macrophages, leading to TNF-α and IFN-α release as well as enhanced antifungal activity by plasmacytoid dendritic cells (Loures et al., 2015).

Finally, no host receptor has yet demonstrated for chitin, the inner component of the *Aspergillus* cell wall. The immune response to chitin is discordant and the exact mechanisms determining its inflammatory signature are poorly understood. It was shown to have pro-inflammatory as well as anti-inflammatory properties depending on the presence of costimulatory pathogen-associated molecular patterns and immunoglobulins (Becker et al., 2016). Its role is context specific since its recognition and ability to engage with receptors depends on cell-type, concentration and particle size (Da Silva et al., 2009). However, it seems most of the studies associate chitin with a predominantly type-2 response (Snarr et al., 2017).

### Cell Wall as an Antifungal Target

As it has been described before, echinocandins drugs are antifungal compounds that target the β-1,3-glucan synthesis of the cell wall (Aguilar-Zapata et al., 2015). However, due to the limited antifungal activity of these drugs against *Aspergillus* spp., echinocandins compounds are used only as an alternative or a salvage therapy for the treatment of invasive aspergillosis when the first line therapy with azole drugs fails (Aruanno et al., 2019). It is noteworthy that a new antifungal drug called ibrexafungerp (SCY-078) has a broad *in vitro* and *in vivo* activity against a broad spectrum of *Aspergillus* species (Ghannoum et al., 2018).

Currently, there are no *Aspergillus* licensed vaccines to protect humans from aspergillosis (Levitz, 2017). Recently, the Cassone group developed a conjugate of β-1,3-D-glucan in the form of laminarin and the diphtheria toxoid CRM197. Carbohydrate antigens are poorly immunogenic so conjugation with a protein carrier greatly boosts specific antibody responses, protecting in this case against *A. fumigatus* and *C. albicans* (Torosantucci et al., 2005). Additionally, purified cell wall glycans have been used as immunogens through intranasal vaccination with α- and β-1,3-D-glucans but not with galactomannan (Bozza et al., 2009). Given the high morbidity and mortality associated with aspergillosis, much work still needs to be done if vaccines against this pathogen are to become a real option.

## Conclusion

The fungal cell wall represents an organelle whose composition plays a crucial role in cell viability, morphology and protection against different stressors. Within the fungal kingdom, there is heterogeneity in the composition of the cell wall with species that have unique characteristics that differentiate them from other fungi. The synthesis of the main components of the cell wall is carried out by different genes, among which the *FKS1*, *AGS1*, and *CHS* genes stand out, although there are thousands of genes involved in synthesis, signaling and cell wall assembly. Throughout this review, we have discussed how the different components of the cell wall play an important role in the virulence of these pathogens and how the cell wall interacts with the host’s immune system. Mutants of genes involved in the synthesis of different wall components have shown loss of virulence in animal models in *Candida* species. The fungal cell wall remains the most attractive target for the next generation of antifungal drugs. Although it is true that in the last decade, the biology of the fungal cell wall has been studied in depth, many questions remain unanswered requiring additional studies.

## Author Contributions

NT-C, RG-R, HO, and JR wrote the original draft of the manuscript. HO designed the scheme. JR reviewed the English language of the manuscript. NT-C supervised the study.

## Conflict of Interest

The authors declare that the research was conducted in the absence of any commercial or financial relationships that could be construed as a potential conflict of interest.
